# Supplementary material for: Identification of a Novel Oncogenic Fusion Gene SPON1-TRIM29 in Clinical Ovarian Cancer That Promotes Cell and Tumor Growth and Enhances Chemoresistance in A2780 Cells
Source: Int J Mol Sci. 2022 Jan 8;23(2):689. doi: 10.3390/ijms23020689 (PMC8776205; doi:10.3390/ijms23020689)
Supplement: Supplementary file 1 [file ijms-23-00689-s001.zip › ijms-1510837-supplementary.pdf]

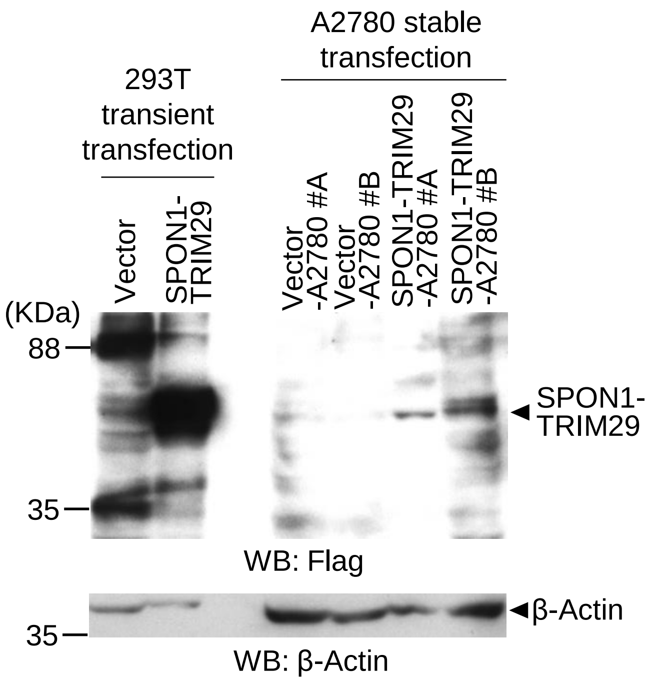

Figure S1. A2780 cells stably transfected with *SPON1-TRIM29* (SPON1-TRIM29-A2780 #A and #B) or empty vector (Vector-A2780 #A and #B). Cells were stably transfected with a Flag-tagged *SPON1-TRIM29* expression vector or an empty vector, and the transformants were generated under G418 selection. 293T cells were transiently transfected with these vectors. Cell lysates were analyzed by Western blotting probed with anti-Flag antibody. β-Actin was used as a loading control for Western blotting.

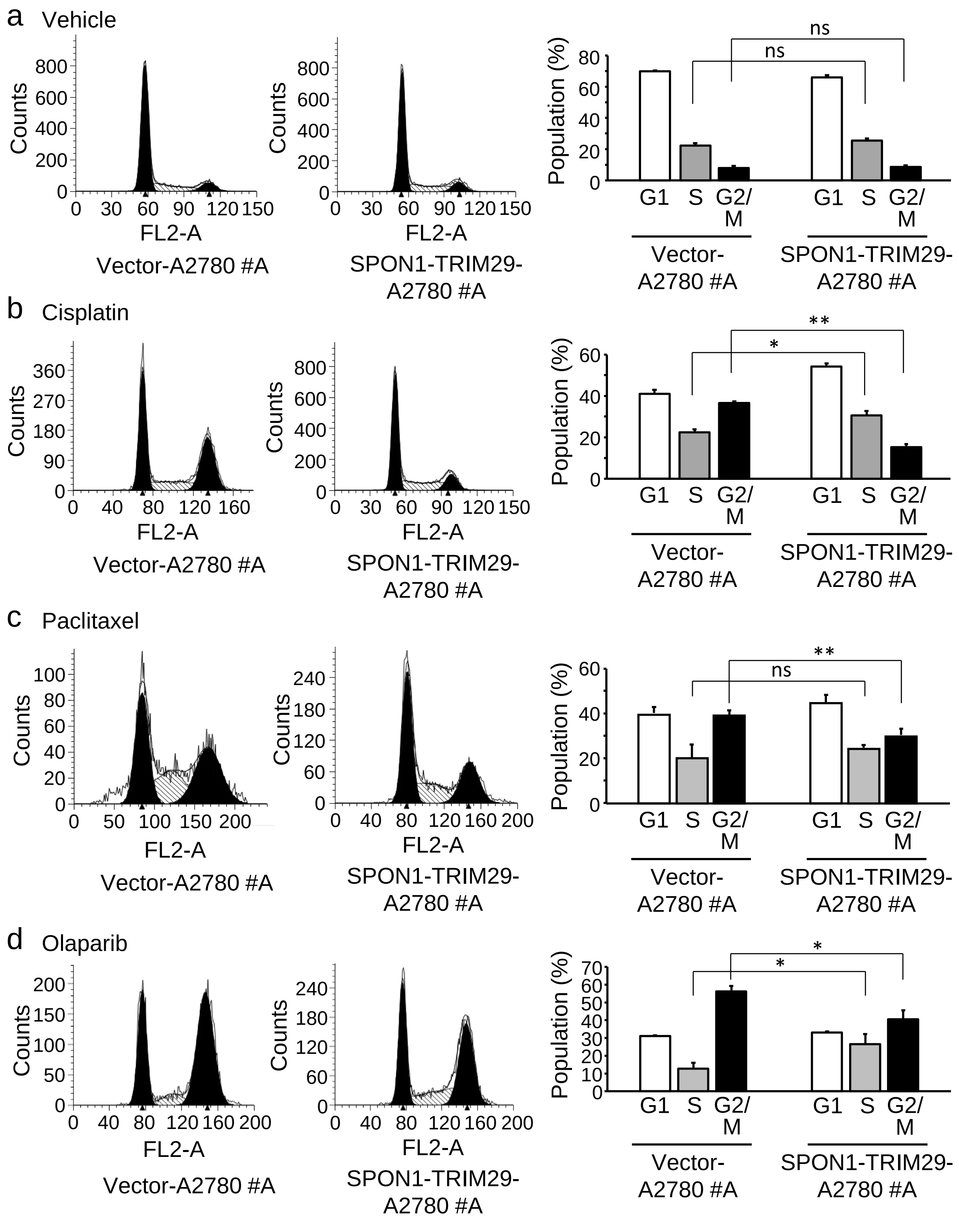

Figure S2. Stimulation of cell-cycle progression in SPON1-TRIM29-A2780 cells under cancer drugs treatment. SPON1-TRIM29-A2780 #A and Vector-A2780 #A cells were treated with vehicle (a), 1 μM cisplatin (b), 5 nM paclitaxel (c), or 2 μM Olaparib (d) for 5 days, stained with propidium iodide, and then subjected to FACS analysis. The percentages of cells in the G1, S, and G2/M phases were calculated using the CellQuest software. Data are presented as means ± SD (*n* = 3). **P* < 0.05; ***P* < 0.01, Student's *t*-test. ns, not significant.

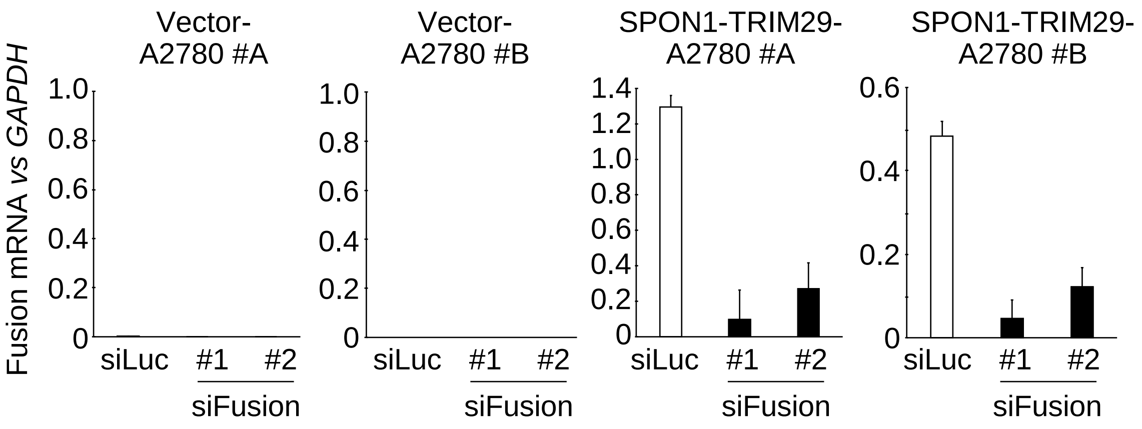

Figure S3. SPON1-TRIM29 knockdown by siRNA. SPON1-TRIM29- and Vector-A2780 cells were treated with siRNAs specific for *SPON1-TRIM29* joint sequence. RT-qPCR analysis of *SPON1-TRIM29* expression was performed. The mRNA levels of *SPON1-TRIM29* fusion transcript are normalized with *GAPDH*. Data are presented as means ± SD (n = 3).

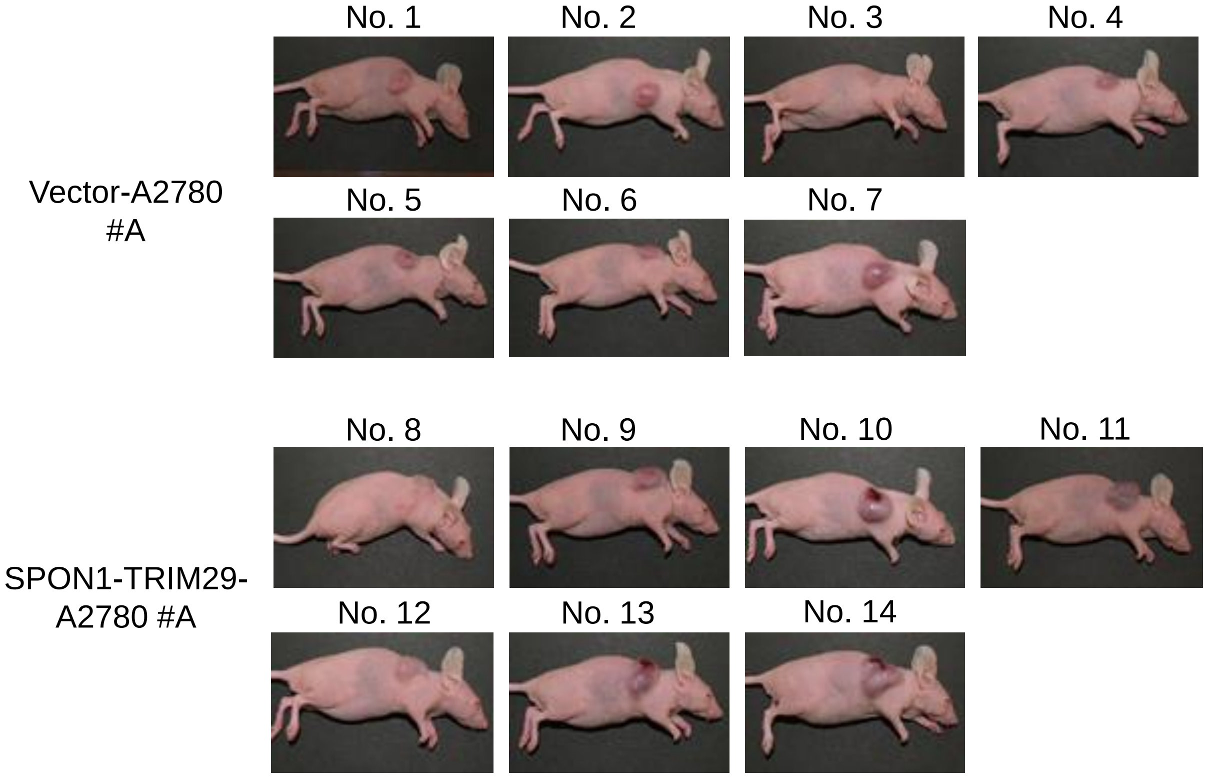

Figure S4. Increased tumor volumes of SPON1-TRIM29-A2780 cells in athymic mice. Female athymic mice were subcutaneously injected with SPON1-TRIM29-A2780 #A or Vector-A2780 #A cells. Images of the tumor-bearing mice at end point are shown.

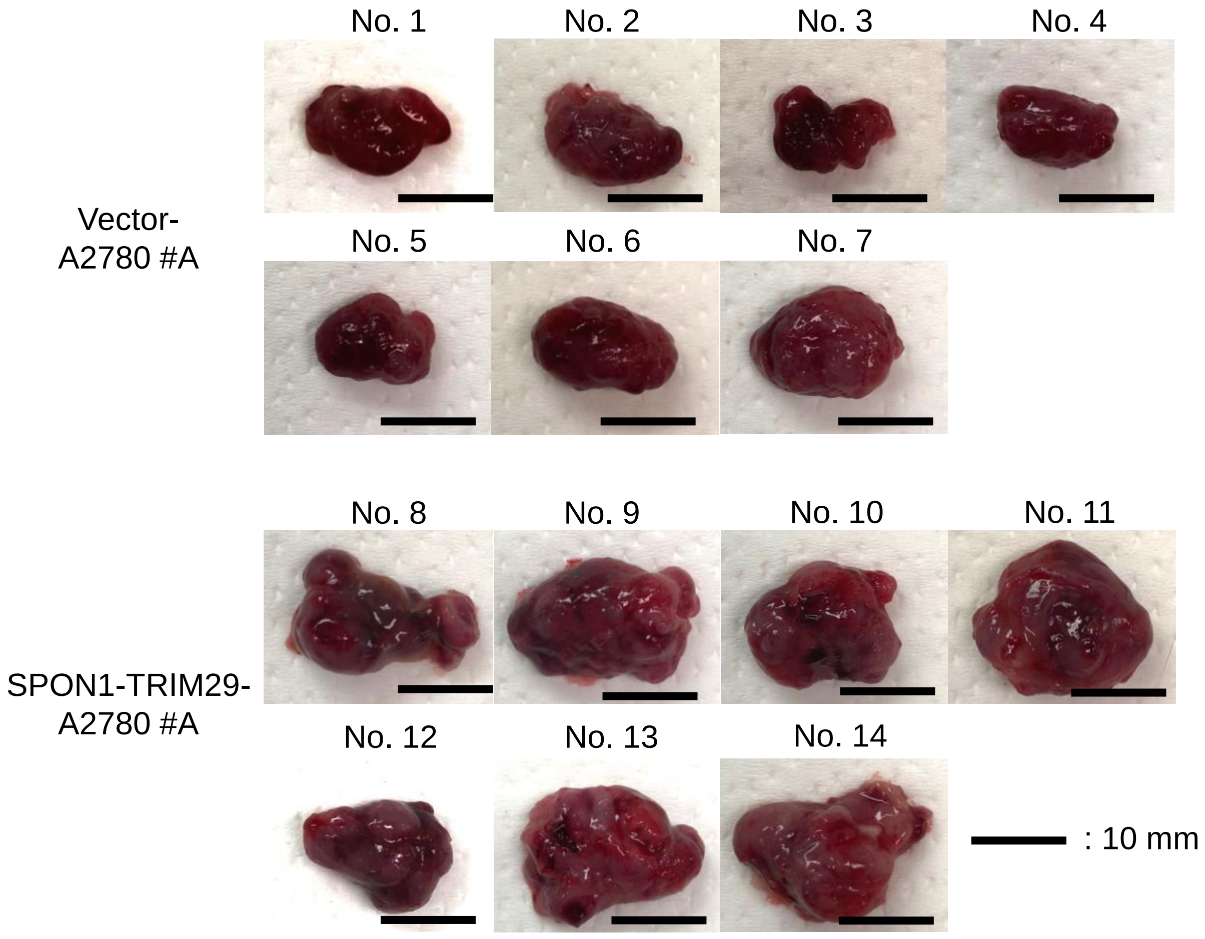

Figure S5. Dissected tumors from SPON1-TRIM29-A2780 #A- and Vector-A2780 #A-bearing mice shown in Figure S4 are presented.

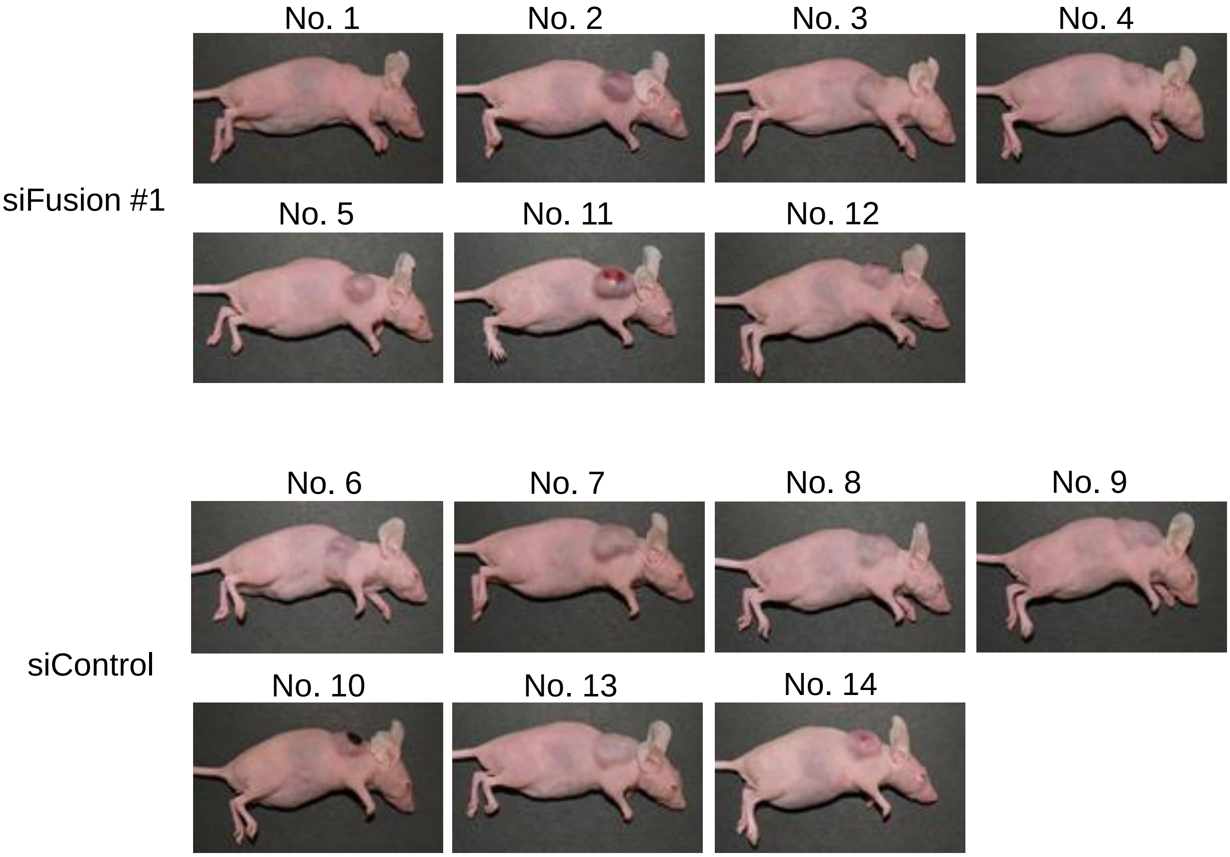

Figure S6. Inhibition of tumor formation in athymic mice after SPON1-TRIM29 siRNA treatment. Female athymic mice were subcutaneously inoculated with SPON1-TRIM29-A2780 #A cells and then administrated with control siRNA (siControl) or SPON1-TRIM29 siRNA (siFusion #1) every 3 days. Images of the tumor-bearing mice at end point are shown.

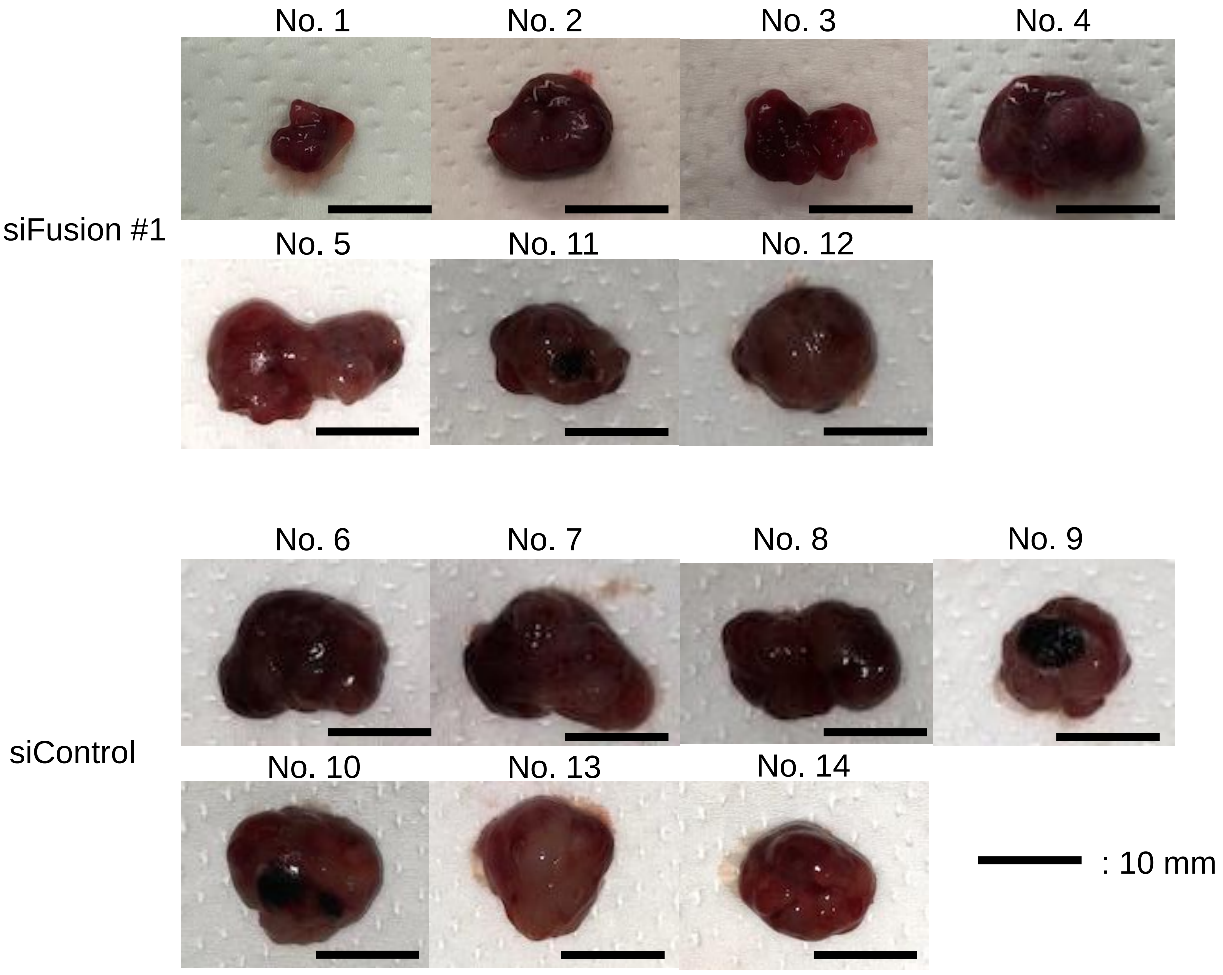

Figure S7. Dissected tumors from mice administrated with control siRNA (siControl) or SPON1-TRIM29 siRNA (siFusion #1) shown in Figure S6 are presented.
